# [Corrigendum] Antitumor effects of dioscin in A431 cells via adjusting ATM/p53-mediated cell apoptosis, DNA damage and migration

**DOI:** 10.3892/ol.2026.15664

**Published:** 2026-05-21

**Authors:** Peng Wang, Chun Wang, Chunying Liu

Oncol Lett 21: 59, 2021; DOI: 10.3892/ol.2020.12321

Following the publication of the above paper, it was drawn to the authors' attention by an interested reader that, regarding the Transwell assay data shown in Fig. 1E on p. 3, the ‘Dio2.9 μM’ and ‘Dio5.8 μM’ data panels contained an overlapping section, such that these data were apparently derived from the same original source where they were intended to have shown the results of differently performed experiments.

Upon consulting their original data, the authors have realized that the mistakes made in this figure were due to a cut-and-paste error. The revised version of Fig. 1, now showing the correct data for the ‘Dio2.9 μM’ and ‘Dio5.8 μM’ data panels in Fig. 1E, is shown on the next page. Note that this error did not affect the overall scientific conclusions reported in the article. The authors are grateful to the Editor of *Oncology Letters* for allowing them the opportunity to publish this Corrigendum, and all the authors agree with its publication. They also thank the reader of the article for drawing this matter to their attention.

## Figures and Tables

**Figure 1.**
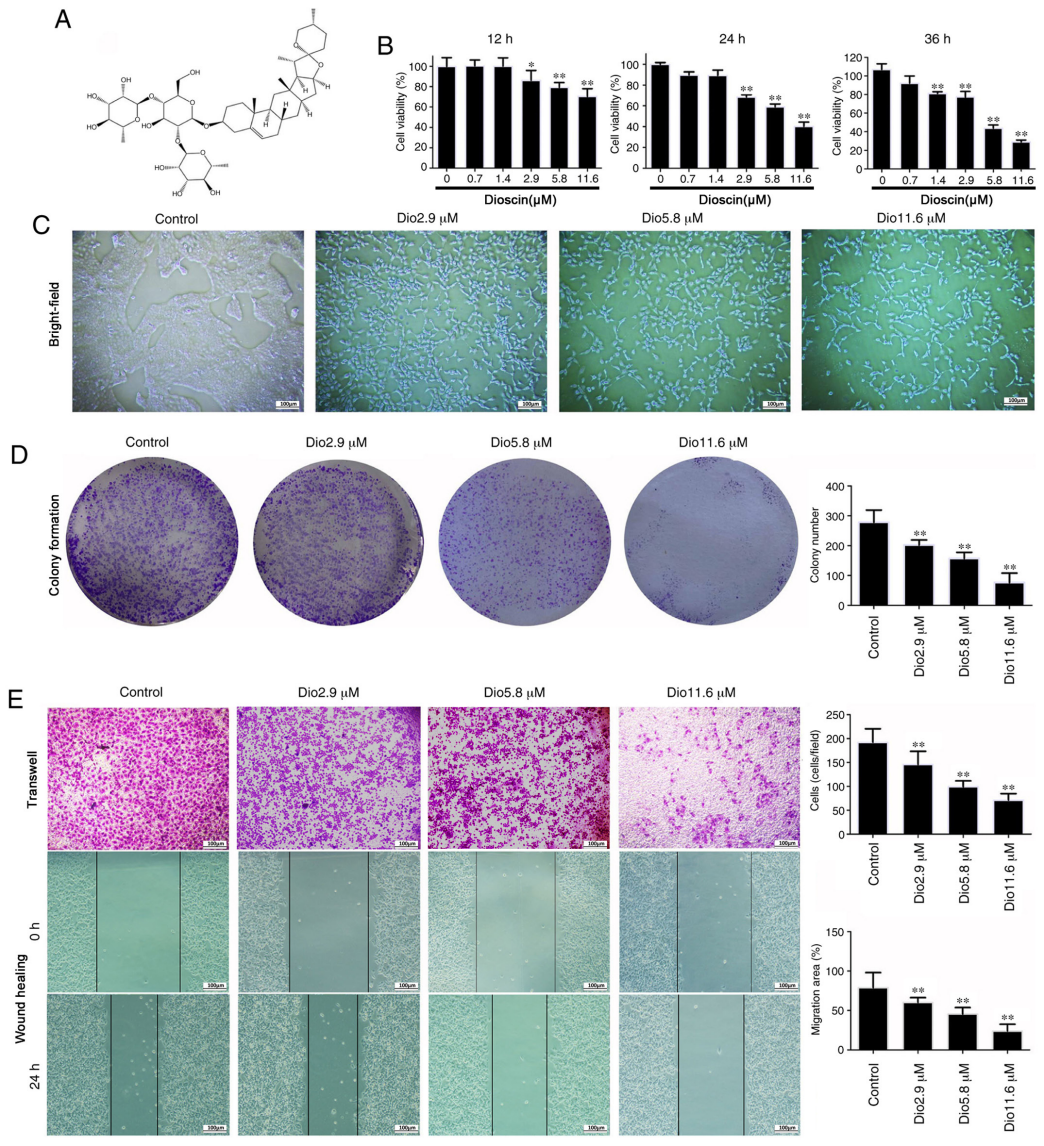
Inhibitory effects of dioscin on the viability, colony formation and migration of A431 cells. (A) Chemical structure of dioscin. (B) Effects of dioscin on the viability of A431 cells detected via MTT assay. (C) Representative morphological images of cells treated with different concentrations of dioscin (2.9, 5.8 and 11.6 µM) for 24 h. (D) Effects of dioscin treatment (2.9, 5.8 and 11.6 µM) for 24 h on colony formation in A431 cells. (E) Effects of dioscin treatment (2.9, 5.8 and 11.6 µM) for 24 h on migratory and invasive properties of A431 cells. Scale bar, 100 µm. Data are presented as the mean ± SD (n=5). *P<0.05 and **P<0.01 vs. control. Dio, dioscin.

